# Revealing the Nanoscale Morphology Changes of Polymer Electrolyte Membrane Fuel Cell Catalyst Layers due to Conditioning

**DOI:** 10.1002/advs.77009

**Published:** 2026-08-10

**Authors:** Spencer Lytle, Harsharaj B. Parmar, Jian Wang, Shohei Yamashita, Aimy Bazylak

**Affiliations:** ^1^ Bazylak Group, Department of Mechanical & Industrial Engineering, Faculty of Applied Science and Engineering University of Toronto Toronto Ontario Canada; ^2^ Canadian Light Source Inc. Saskatoon Saskatchewan Canada; ^3^ Photon Factory, Institute of Materials Structure Science High Energy Research Organization Tsukuba Ibaraki Japan

**Keywords:** catalyst layer, electrochemical conditioning, membrane dynamics, scanning transmission X‐ray spectroscopy, structural degradation

## Abstract

We applied ex situ and in situ scanning transmission X‐ray microscopy (STXM) to reveal the structural degradation of polymer electrolyte membrane (PEM) fuel cell catalyst layers due to conditioning and thermal heating of the catalyst coated membrane (CCM). For electrochemically conditioned samples, a homogeneous ionomer‐to‐carbon‐black weight ratio distribution, as quantified by STXM, was observed along the cathode catalyst layer, in contrast to a heterogeneous ionomer‐to‐carbon‐black weight ratio distribution for the pristine cathode catalyst layer. The homogeneous ionomer‐to‐carbon‐black weight ratio, as quantified by STXM, is attributed to the redistribution of ionomer and carbon black into spatially favored positions during operation, likely driven by the loss of structural cohesion within the catalyst layer. However, a heterogeneous porosity distribution was seen across conditioned catalyst layers, which we attributed to pore enlargement that resulted from the incipience of liquid water during conditioning. Furthermore, for the first time, we report real‐time dynamic nanoscale contraction (up to 2.8%) of a conditioned PEM fuel cell membrane subjected to dry and heated conditions. The damage incurred by the catalyst layer from conditioning manifested via the susceptibility of the CCM to tearing during ultramicrotomy, and further worsening of this damage was observed when the membrane was subjected to drying and contraction.

## Graphical abstract

We apply ex situ and in situ scanning transmission X‐ray microscopy to reveal the structural degradation of polymer electrolyte membrane fuel cell catalyst layers due to conditioning and thermal heating of the catalyst coated membrane. We observe a redistribution of the ionomer and carbon‐black in the catalyst layer, as well as nanoscale membrane contraction accompanying unprecedented cyclic catalyst layer damage.

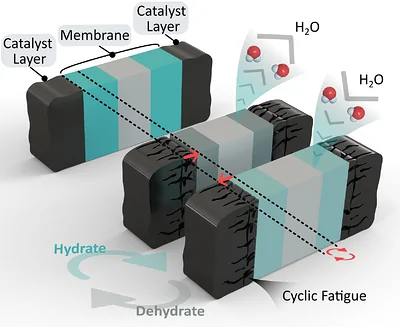

## 1 Introduction

PEM fuel cells are a promising electrochemical technology that efficiently converts hydrogen gas into dispatchable electricity with zero local emissions [1]. Specifically, PEM fuel cells offer clean, high‐energy density power units that are essential for facilitating a cost‐effective transition toward sustainable electrification [2]. However, PEM fuel cells contain high‐cost platinum metal‐based catalyst layers [3] that are susceptible to severe degradation with operation [4, 5, 6]. As a result, PEM fuel cell commercialization is primarily hindered by its degrading electrochemical efficiency, which results in poor longevity and high operating costs compared to alternative renewable power sources.

In a PEM fuel cell, hydrogen and oxygen gas are delivered to the anode and cathode, respectively, through grooved flowfields placed parallel to the catalyst layers [7]. The channel grooves (0.5 – 1.5 mm) [8] of the flowfields are designed to diffuse the reactant gases toward the reaction sites in each catalyst layer. Moreover, a carbonized porous media called a gas diffusion layer (GDL, see Abbreviations Table 1) is positioned between the flow field and catalyst layer to result in a homogeneous distribution of reactants at the catalyst layer interface [9]. The microporous GDL is essential for homogeneously directing the high velocity reactants in the flowfields toward the catalyst layer nanostructure, necessary for the efficient utilization of fuel. At the anode catalyst layer, the platinum‐based catalyst facilitates the thermodynamically favorable hydrogen oxidation reaction (HOR), where hydrogen is dissociated into electrons and protons [10]. Protons can migrate through the electrically insulative proton exchange membrane (located adjacent to the anode catalyst layer) toward the cathode catalyst layer (located across the membrane, collectively forming the CCM). Simultaneously, the sluggish oxygen reduction reaction (ORR) combines the resulting electrons (delivered through an external circuit) and protons (delivered through the membrane) with oxygen gas in the cathode, producing water and heat as the only byproducts [11].

**TABLE 1:** Table of Abbreviations.

Catalyst Coated Membrane	CCM
Gas Diffusion Layer	GDL
Hydrogen Oxidation Reaction	HOR
Microporous Layer	MPL
Near Edge X‐ray Absorption Fine Structure	NEXAFS
Open Circuit Voltage	OCV
Optical Density	OD
Perfluorosulfonic Acid	PFSA
Polyethylene Naphthalate	PEN
Polymer Electrolyte Membrane	PEM
Polystyrene	PS
Polytetrafluoroethylene	PTFE
Region of Interest	ROI
Relative Humidity	RH
Scanning Transmission X‐ray Microscopy	STXM
Spectromicroscopy	SM
Standard Liters per Minute	SLM
Transmission Electron Microscopy	TEM

Each catalyst layer consists of catalyst‐coated carbon black, proton‐conducting polymer (ionomer), and void space that facilitates the electrochemical redox reactions (Figure 1). Carbon black nanoparticles [12] (10‐100 nm) provide structural support to the sub‐10 nm catalyst nanoparticles [13] while facilitating electrical conductivity through each catalyst layer. Conversely, the perfluorosulfonic (PFSA) acid‐based ionomer (Nafion) [14] in the catalyst layer facilitates the conduction of protons through the stochastic network of catalyst‐coated carbon black and void space. Proton conduction is enabled by the hopping of H^+^ ions between the hydrophilic sulfonic acid groups (SO_3_H) terminating the sidechains of the PFSA‐based ionomer (with a polytetrafluoroethylene (PTFE) backbone) [15]. This mechanism requires the precise orientation of sulfonic acid groups to facilitate effective proton transfer. However, an ineffective distribution of ionomer can hinder this process, restricting proton migration [16, 17]. In the presence of liquid water, proton conduction is significantly improved due to the addition of the Grotthuss mechanism (proton hopping through water) and vehicular mechanism (proton transfer through hydronium (H_3_O^+^) ions) [18]. One common method to ensure hydration of the membrane and ionomer in the catalyst layer is electrochemical conditioning of the CCM [19, 20]. Electrochemical conditioning is typically performed after the cell assembly to enhance the performance of the cell without subjecting the CCM to accelerated degradation. In addition, conditioning serves as a mechanism to remove catalyst impurities and accelerate the formation of triple phase boundaries through the catalyst layer [19]. However, the delivery of hydrogen and oxygen gas to their respective HOR or ORR active sites is limited by the aggregation of ionomer, carbon black, catalyst metal, and liquid water during conditioning. Although the optimized dispersions of ionomer and carbon black while manufacturing the CCM are vital for improved PEM fuel cell performance, optimizing mass transport pathways is critical for the effective removal of liquid water generated from the ORR [21]. The accumulation of excess liquid water in the cathode catalyst layer during operation (specifically for low‐temperature PEM fuel cells (below 100°C) operating at high current densities) [22] can inhibit electrochemical efficiency by flooding the catalyst active sites [21, 23, 24, 25, 26, 27, 28]. Thus, a delicate balance of mitigating performance degradation due to flooding while ensuring adequate CCM hydration results in a critical optimization challenge for next‐generation PEM fuel cell designs.

**FIGURE 1:**
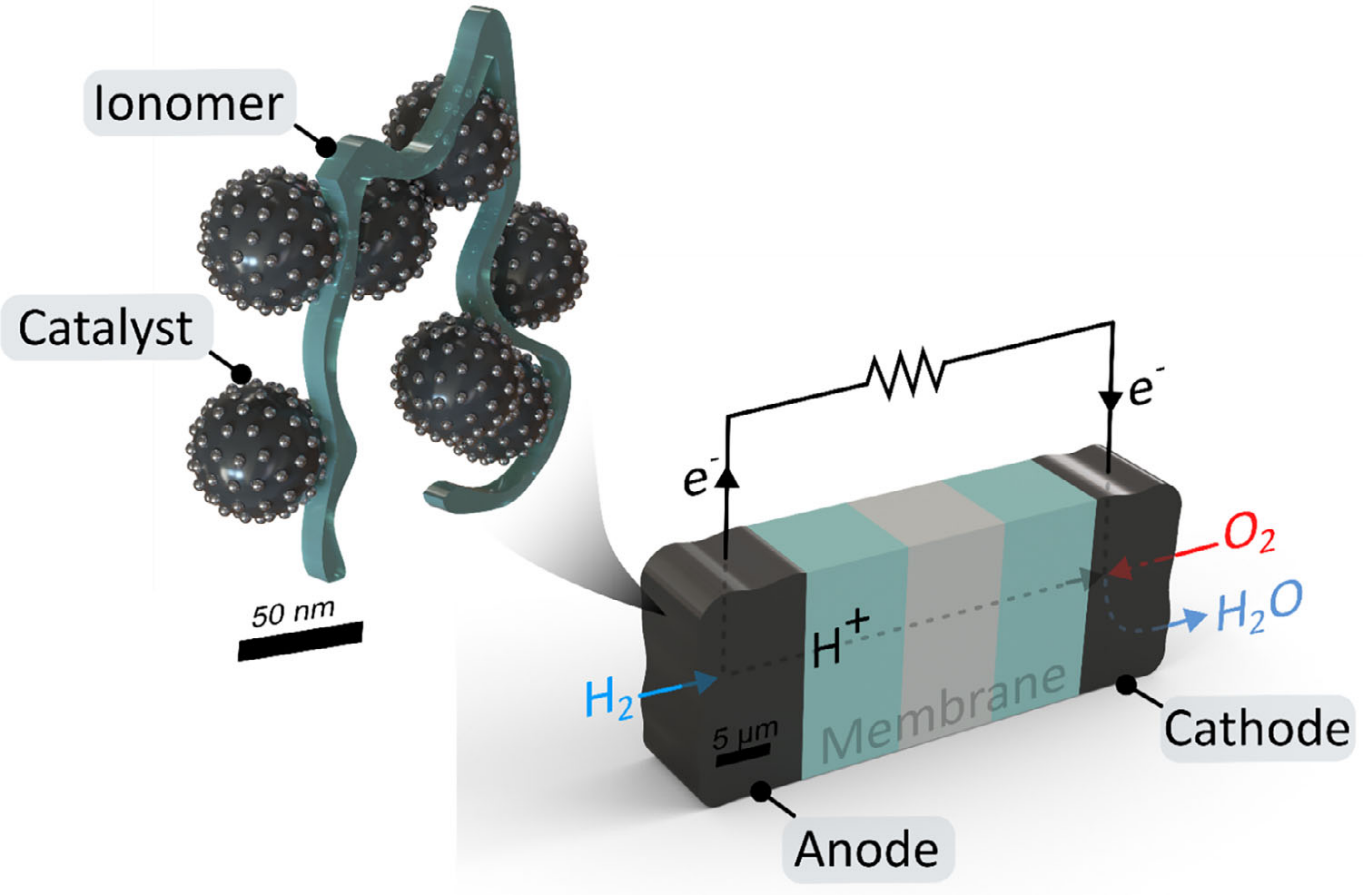
Depiction of a CCM and catalyst nanostructure for a PEM fuel cell.

Furthermore, the literature has vastly explored methods to mitigate liquid water flooding in PEM fuel cells through optimizing flowfields [29, 30, 31, 32, 33], GDLs [34, 35, 36, 37], and catalyst layers [38, 39, 40, 41]. However, the multiscale diffusion lengths from the catalyst layer to the GDL [27] result in a multivariable design challenge to optimize water management. Despite the complexity of the catalyst layer nanostructure [27], many groups have attempted to optimize and fabricate state‐of‐the‐art catalyst layer designs. For example, Eikerling et al. found that altering the wetting angle of the cathode catalyst layer has pronounced impacts on the current densities that initiate flooding (critical current densities) [42]. Controlling the cathode catalyst layer wettability during fabrication can lead to effective water management that saturates the primary pore space (pores within ionomer/carbon agglomerates) [43] and leads to an accessible secondary pore space (pores outside ionomer/carbon agglomerates) [43]. Additionally, Wan et al. explored the effects of cathode wettability gradients on the performance of the cell, and revealed a significant improvement in the mass transport regime [44]. As a result of the induced capillary pressure difference across the wettability gradient, the removal of liquid water toward the microporous layer (MPL) was significantly improved. Furthermore, Mohseninia et al. employed high‐resolution neutron tomography to map the effects of catalyst layer perforation (macropores) on the distribution of water [45]. It was revealed that perforated layers promoted the formation of liquid water beneath the land regions and enabled a superior liquid water removal under the channels. Similarly, Lee et al. explored the effects of grooved electrodes on the improvement in PEM fuel cell performance which led to significant reductions in oxygen diffusion resistances [46]. The fabricated grooves (1‐2 µm width and 3‐6 µm depth) particularly improved performance in higher ionomer‐to‐carbon‐black (I/C) weight ratios, attributed to the rapid O_2_ diffusion through the grooves while facilitating effective H^+^ migration in ionomer peaks.

Furthermore, the effects of hydration on the catalyst layer nanostructure have been examined through liquid water/ionomer interactions. For relative humidity (RH) values greater than 60%, Kaneko et al. observed a reduction in pore space using density functional theory (DFT) simulations [47]. Similarly, membrane swelling and shrinking due to water absorption and desorption has been extensively investigated. Alavijeh et al. found that hydrocarbon and reinforced PFSA ionomer exhibited greater membrane shrinkage than swelling, resulting in elevated internal stresses that increased with hydration cycling [48]. Furthermore, the addition of catalyst layers onto PFSA membranes favorably reversed the hydration‐related swelling/shrinkage, since they acted as an additional support to the PTFE‐reinforced membrane backbone. Moreover, Ge et al. observed membrane shrinkage at elevated temperatures, current densities, and inlet relative humidities through *operando* X‐ray radiography [49]. The membrane shrinkage was attributed to reduced local RH (due to an increase in local temperature) at the CCM during operation at high current densities [49]. Despite attempts to mitigate water‐induced performance degradation during PEM fuel cell operation, the nanostructural behaviors of the cathode catalyst layer during operation are still unclear in the literature. Specifically, the effects of electrochemical activity on the redistribution of ionomer, carbon black, and void space need to be investigated to design efficient and cost‐effective platinum‐loaded catalyst layers.

Thus, in this work, we employed ex situ and in situ STXM to quantify the effects of temperature and electrochemical conditioning on the nanostructure of PEM fuel cell CCMs. Particularly, we quantified the I/C weight ratio and porosity distributions in conditioned and pristine cathode catalyst layers to reveal the morphological changes attributed to the enhanced performance in conditioned CCMs. Moreover, we elucidated the effects of temperature on conditioned CCMs at a 60 nm spatial resolution and provide a novel characterization of membrane and catalyst layer movement with time. Our results provide a deeper understanding of catalyst layer nanostructures for the design of next‐generation, high‐performance CCMs.

## 2 Materials and Methods

In this work, electrochemical conditioning (break‐in) of commercial CCMs was conducted using a custom, single cell PEM fuel cell. Conditioned and pristine CCMs were prepared for ex situ and in situ imaging via ultramicrotomy, where each CCM was supported with polystyrene (PS) and cut into 100 nm‐thick (ex situ) and 300 nm‐thick (in situ) through‐plane sections. Separate samples of pristine and conditioned CCMs were used for ex situ and in situ analyses. Subsequently, to investigate the effects of electrochemical conditioning on the distributions of ionomer, carbon black, and porosity, ex situ imaging at the carbon K‐edge was performed using STXM and near edge X‐ray absorption fine structure (NEXAFS) spectroscopy. Finally, pristine and conditioned CCMs were imaged in situ at the fluorine K‐edge at 25°C and 60°C using STXM and NEXAFS spectroscopy to investigate the effects of temperature on the catalyst layer nanostructures.

### 2.1 Materials and MEA Preparation

The effects of temperature and electrochemical conditioning on PEM fuel cell CCMs were investigated using in situ spectromicroscopy (SM). Commercial Nafion HP CCMs with an active area of 0.68 cm^2^ were obtained from Ion Power Inc., New Castle, DE, USA, with a platinum loading of 0.3 mg Pt/cm^2^ at the anode and cathode catalyst layers. The proton‐conducting membrane was composed of a PTFE backbone for structural support, sandwiched by PFSA ionomer layers (Nafion).

To evaluate the effects of electrochemical conditioning on a CCM, a custom PEM fuel cell fixture (Figure 2a) was used to condition the commercial Nafion HP CCM via voltage cycling. Parallel graphite flow fields (8 mm in length) with square profile lands and channels (0.5 x 0.5 mm) were used for co‐flow gas delivery/water removal to/from the GDLs. Water‐treated (10% PTFE) Toray carbon paper 060 (Fuel Cell Store, Bryan, TX, USA) GDLs, with a total nominal thickness of 250 µm (190 µm GDL and 60 µm MPL), were used at both the anode and cathode sides. After torquing the cell assembly with six pairs of M4 bolts to 15 in·lbs, the original thicknesses of the GDL assembly (250 µm with GDL and MPL) was compressed by 40% using 150 µm‐thick polyethylene naphthalate (PEN) gaskets. After assembly, a standard break‐in protocol from Scribner Associates Inc. was conducted to condition the CCM, controlled by an 850e Fuel Cell Test Station (Scribner Associates, Inc.) [50]. After reaching steady state conditions controlled using the test station (60°C, 80% RH, and 0.5 standard liters per minute (SLM) of air and hydrogen gas (AlphaGAZ1, > 99.99%)), the cell was subjected to potential holds at open circuit voltage (OCV), 0.6 V, and 0.3 V for 60 s each. Thereafter, the cycle was repeated 60 times to complete the conditioning process for a total of 180 min.

**FIGURE 2:**
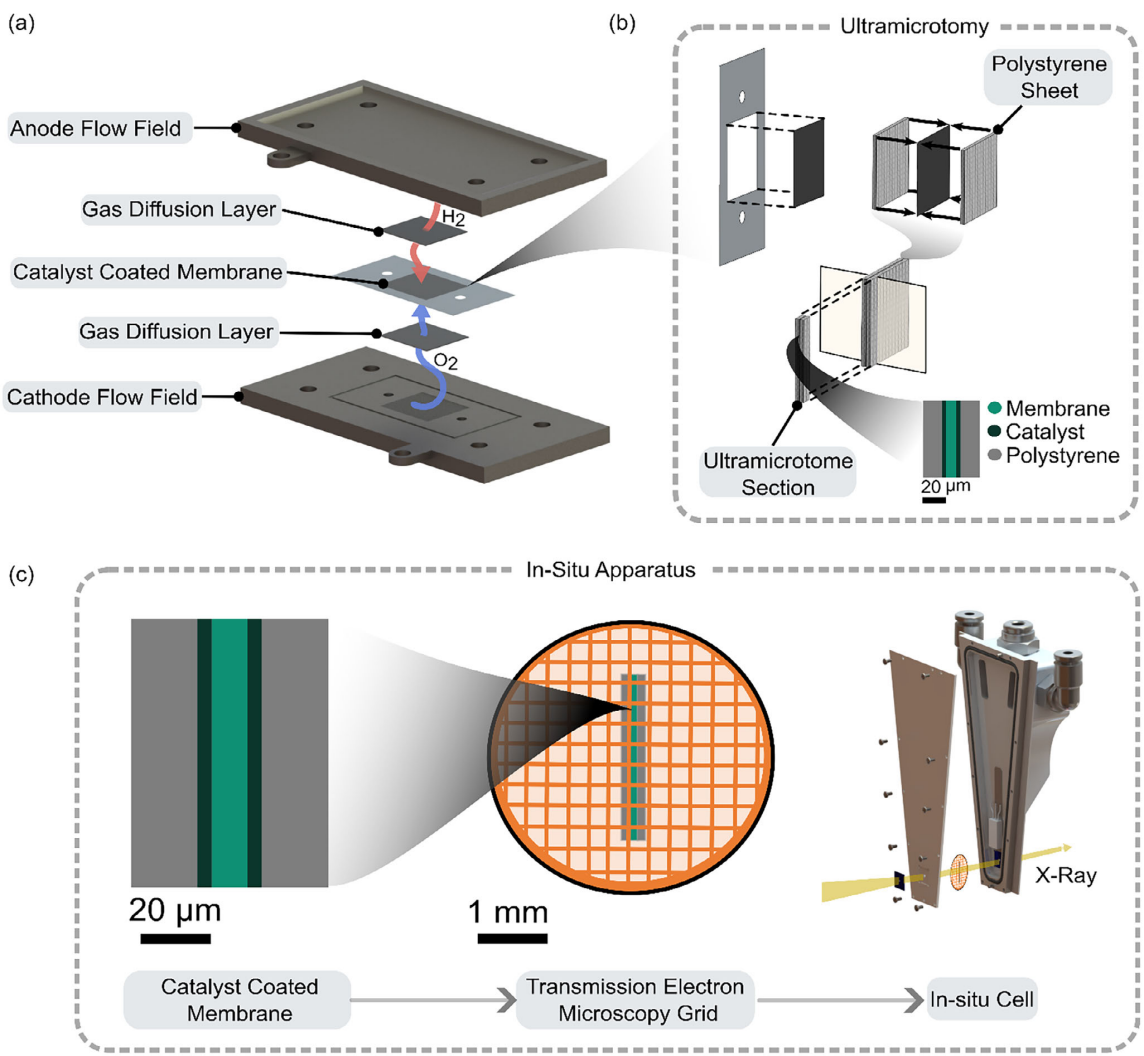
Schematic of the experimental apparatus to investigate the impact of conditioning and temperature on PEM fuel cell CCMs. (a) Exploded view of a PEM fuel cell used for electrochemical conditioning, revealing the flowfields, GDLs, and CCM. (b) Shows the extraction of the CCM from a gasket for ultramicrotomy, and (c) depicts the custom in situ cell apparatus with a mounted TEM grid for STXM imaging.

### 2.2 Ex Situ STXM

Each CCM sample was conditioned using the custom, single‐cell PEM fuel cell (Figure 2a), and subsequently prepared for STXM imaging via ultramicrotomy, as shown in Figure 2b. Thereafter, the CCMs were sandwiched between PS sheets (C_8_H_8_ with a density of 1.05 g/cm^3^) [51] and cut into 100 nm‐thick and 300 nm‐thick through‐plane sections using an ultramicrotome (Leica Ultracut UCT ultramicrotome, Leica Microsystems, Wien, Austria) equipped with an Ultra 45° DiATOME knife (Ted Pella Inc, USA). Finally, sectioned CCMs were placed onto formvar‐coated transmission electron microscopy (TEM) grids for STXM imaging. Using a similar approach to Susac et al., pure Vulcan XC‐72R carbon black powder, with a nominal diameter [52] of 20‐50 nm, was acquired from Fuel Cell Store (Bryan TX, USA) as a reference material for the detection of carbon black in the catalyst layer [53]. The pure carbon black powder was dispersed in a water‐based solvent and deposited onto a TEM grid for NEXAFS spectroscopy at the carbon K‐edge. The ultramicrotomy thicknesses of the CCMs (100 nm for ex situ and 300 nm for in situ) were kept adequately larger than the carbon black particle sizes to preserve the carbon black distribution in the sectioned samples.

To quantify the effects of electrochemical conditioning on the distributions of ionomer, carbon black, and porosity in a PEM fuel cell catalyst layer, ex situ STXM imaging was performed at the BL‐19A/B beamline at the Photon Factory in Tsukuba, Japan [54]. Prior to imaging, the STXM chamber was evacuated and back filled with pure helium to ensure an inert environment during imaging. Moreover, the vacuum environment additionally prevents X‐ray interference with moisture retained within the conditioned samples. Due to the limited field of view and acquisition time inherent to synchrotron‐based STXM imaging [53], STXM imaging with NEXAFS spectroscopy was conducted on three regions of interest (ROIs) (15 µm x 5 µm) for the 100 nm‐thick pristine and conditioned samples, in addition to two 10 µm x 10 µm ROIs of the carbon black powder samples. Previous STXM studies have shown that STXM can provide an adequate representation of material behaviors using the localized regions of interest [53, 55, 56, 57]. Due to the high carbon concentration of the catalyst layer, 100 nm‐thick sections enabled sufficient X‐ray transmission [53, 58] while mitigating absorption saturation through the carbon‐heavy catalyst layers. Each distinct ROI was strategically positioned to contain regions of the cathode catalyst layer, membrane, and PS region in order to capture the interface between the catalyst layer and membrane. Monochromatic soft X‐rays were focused using a Fresnel zone plate with an outer fringe width of 25 nm to achieve a maximum spatial resolution of 30 nm/pixel. Subsequently, an image was captured at each of 75 distinct photon energies spanning 280 eV to 320 eV (with a maximum energy resolution of 0.3 eV), hereafter referred to as an “energy stack”. We obtained an energy stack for each ROI to characterize the carbon‐containing Nafion ionomer and carbon black species distributed throughout each CCM catalyst layer. To extract the nafion and carbon black distributions from the captured image stack, the stacks undergo extensive post‐processing analyses guided by Marcus [59], Hitchcock [60], and Parmar et al. [61]. The detailed methodology relating to the STXM data processing workflow for this study has been included in the Supporting Information.

### 2.3 In Situ STXM

A custom in situ sample holder (cell) was developed for the SM beamline at the Canadian Light Source (CLS) [62] and was used to control the temperature of the CCM sections during imaging, depicted in Figure 2c. Notably, this cell for STXM imaging is not an electrochemical cell. Electrochemical conditioning was performed prior to the in situ STXM analyses in the PEM fuel cell fixture shown in Figure 2a. In particular, the in situ cell (for STXM) was equipped with a PID controlled cartridge heater, thermocouple, and RH sensor (Zesta Engineering Ltd., Ontario, Canada) to monitor and control the temperature. To maintain cell conditions during imaging, the cell was sealed using a rubber O‐ring to isolate the sample environment from the surrounding STXM chamber. Two silicon nitride windows with a transmission area of 0.25 mm^2^ (0.5 x 0.5 mm) (Norcada Inc., Edmonton, Canada) were used to facilitate a sealed‐transmission path through the in situ apparatus. The 100 nm‐thick windows were aligned and mounted in the in situ sample holder to ensure optimal X‐ray transmission path from the synchrotron X‐ray source to the STXM photon detector. The silicon nitride windows were fixed and sealed to the in situ cell using an epoxy resin. Using the custom in situ STXM cell, pristine and conditioned CCMs (previously conditioned using the PEM fuel cell fixture in Figure 2a) can be analyzed at unique temperature conditions.

To quantify the ionomer volume fraction distributions (calculated using Equation S8) in the pristine and conditioned catalyst layers at 25°C and 60°C, the samples were imaged at the soft X‐ray SM beamline at the CLS synchrotron in Saskatoon, Canada [62]. Prior to in situ imaging, the custom in situ sample holder was mounted into the STXM chamber, and all gas and electrical connections were sealed using epoxy to isolate the sample environment. The STXM chamber was similarly evacuated and back filled with pure helium gas. A PID temperature controller (Zesta Engineering Limited, Mississauga, ON, Canada) operating at 120 V (5 A) was used for temperature control of the in situ sample holder. To prevent interference with the X‐ray beam, an inert environment was maintained in the in situ cell by flowing dry and pure helium gas through the cell at 0.1 SLM using a mass flow controller (MKS, Massachusetts, USA). After steady state temperature was reached, STXM imaging with NEXAFS spectroscopy at the fluorine K‐edge was conducted for two ROIs (15 µm x 5 µm) for the 300 nm‐thick pristine and conditioned samples. As a result of a smaller fluorine concentration in the catalyst layer compared to carbon, 300 nm‐thick sections were deemed optimal to ensure adequate X‐ray transmission and fluorine absorption through the sample. Nonetheless, each ROI similarly encompassed the cathode catalyst layer, membrane, and PS region of each CCM, focused using a 50 nm‐thick outer fringe width Fresnel zone plate (Applied Nanotools Inc., Edmonton, Canada) to achieve a maximum spatial resolution of 60 nm/pixel. Subsequently, energy stacks containing 84 distinct photon energies spanning 680 eV to 720 eV (with a maximum energy resolution of 0.18 eV) were collected at both 25°C and 60°C and 0% RH, enabling the characterization of fluorine‐containing Nafion ionomer in the catalyst layer and membrane.

## 3 Results and Discussion

In this section, the effects of electrochemical conditioning and temperature on PEM fuel cell CCMs are explored. We first visualized the structural degradation of the catalyst layer due to conditioning in Section 3.1, evident through the substantial increase in catalyst layer tearing after ultramicrotomy. Subsequently, we measured the distributions of ionomer and carbon black across pristine and conditioned catalyst layers in Section 3.2, and thereafter the influence of electrochemical conditioning on the porosity distribution (Section 3.3). Finally, we further examined the impact of temperature on conditioned membranes and catalyst layers in Section 3.4 and 3.5, revealing the never‐seen‐before, temperature‐induced degradation prevalent to hydrated CCMs.

### 3.1 Visualizing Catalyst Coated Membrane Nanostructures Using STXM

The nanostructure of pristine and conditioned CCMs was investigated using ex situ STXM. Notably, severe catalyst layer damage was observed in the conditioned catalyst layers (Figure 3b) compared to the pristine samples (Figure 3a). The observed damage in the conditioned CCMs is attributed to changes in the structural cohesion of the catalyst layer nanostructure after electrochemical conditioning. As a result, conditioned CCMs are more susceptible to tearing during the ultramicrotome process, evident by a 30% increase in void space compared to the pristine samples (Figure 3c). This suggests that electrochemical conditioning may strongly influence the distribution of the ionomer, carbon black, and void space that alter the structural cohesion of the conditioned catalyst layer nanostructures (later explored in Section 3.2 and 3.3). As a result, external forces on the CCM during operation, such as the expansion/contraction of the membrane during hydration cycling, are anticipated to degrade the structural properties of the catalyst layer nanostructure over time (later explored in Section 3.4 and 3.5).

**FIGURE 3:**
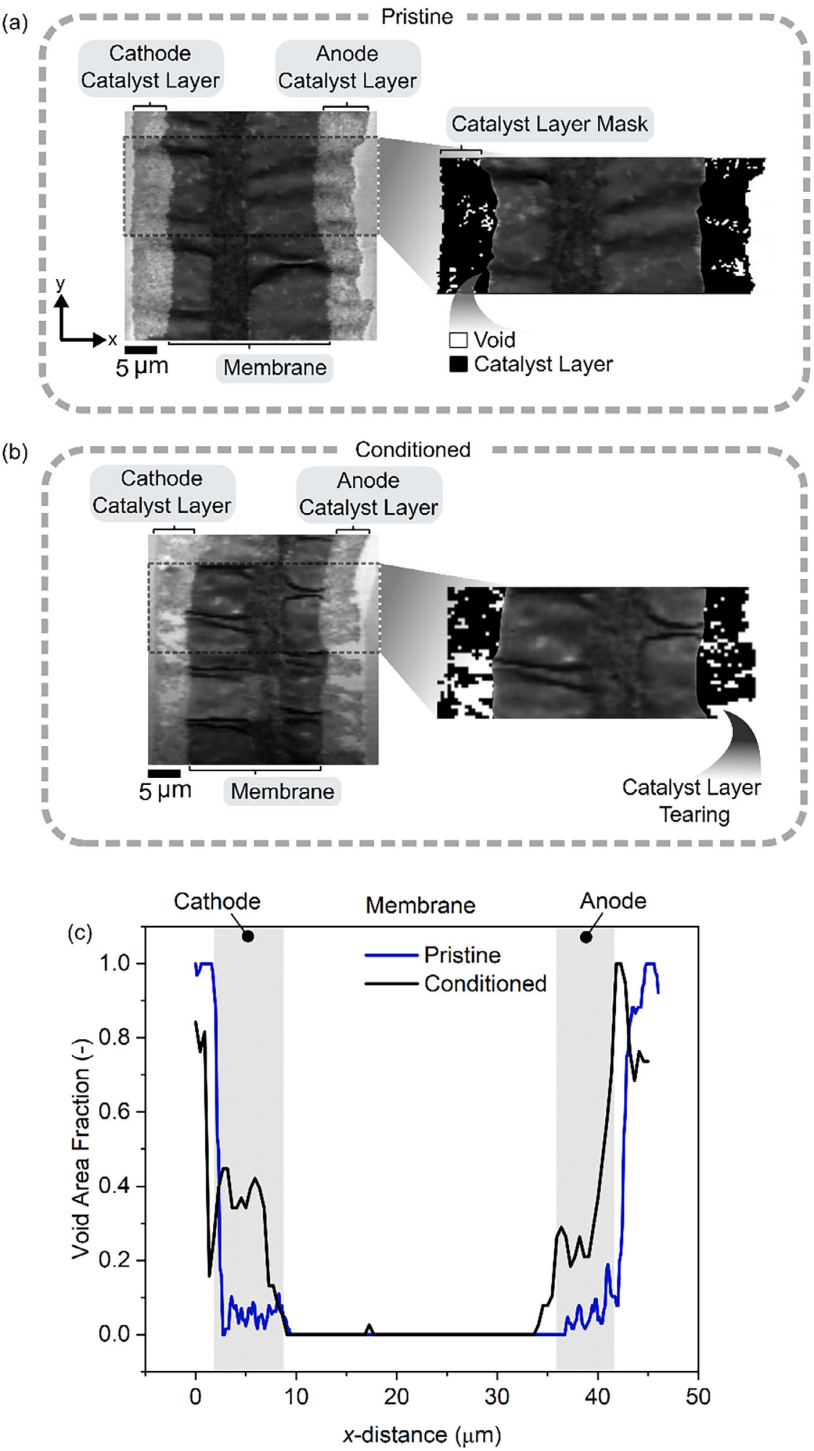
Transmission‐based STXM images of (a) a pristine and (b) a conditioned CCMs at 693 eV, each accompanied by a 15 x 40 µm binarized (masked) section revealing the void space of the respective anode and cathode catalyst layers. The void space area fraction distributions are quantified in (c) for both pristine and conditioned samples, where the conditioned catalyst layers show a higher void space in both catalyst layers.

Furthermore, the observed damage to the conditioned CCMs is attributed to the loss of structural cohesion of the catalyst layer nanostructure during operation. In particular, the loss of structural cohesion is attributed to the swelling and delamination of the ionomer from the carbon‐supported catalyst [63]. The dynamic evolution of water and heat in the cathode catalyst layer during voltage cycling can expand and contract the proton‐conducting ionomer, resulting in the formation of free ionomer as it separates from the platinum nanoparticles (ionomer delamination) [63, 64]. Consequently, we propose that the delamination of ionomer from the platinum nanoparticles compromises the structural cohesion of the catalyst layers, resulting in the heightened tearing observed after ultramicrotomy. However, considering the substantial increase in void area for both the anode and cathode catalyst layers, we assumed that cathode water generation is not solely responsible for catalyst layer structural degradation. During cell compression and voltage cycling, the formation of catalyst layer microcracks [65, 66] may reduce the structural cohesion of the catalyst layer. Collectively, a combination of water and microcrack‐induced structural degradation of the catalyst layer is anticipated to result in pronounced ultramicrotomy damage in conditioned catalyst layers.

### 3.2 Ex Situ STXM Highlights Homogeneous I/C Weight Ratios After Conditioning

Although electrochemical conditioning was observed to structurally degrade the catalyst layer, a significant improvement in the performance of a fuel cell is often seen after employing standard conditioning protocols [19, 20, 67, 68]. By investigating the nanoscale effects of electrochemical activity on the redistribution of the ion‐conducting species, we aimed to reveal the changes in catalyst layer nanostructure that enhance PEM fuel cell performance. Thus, the effects of electrochemical conditioning on the relative distributions of ionomer and carbon black were investigated. Specifically, ex situ STXM was employed to quantify the I/C weight ratio (given by Equation S10) as a function of the relative *x*‐distance across a pristine (Figure 4a) and conditioned (Figure 4b) CCM at three distinct ROIs within the cathode catalyst layers [53]. Our ex situ STXM results demonstrated a clear distinction in the spatial distribution of I/C weight ratio between the pristine and conditioned CCMs. The pristine and conditioned CCMs were each examined using three uniquely separated ROIs. The average I/C weight ratio distribution for pristine CCMs was observed to significantly vary between the three ROIs (1.25, 1.88, and 2.77, Figure 4a). In contrast, the conditioned CCMs (conditioned using a separate batch of the same pristine CCM type) exhibited similar I/C weight ratio averages across three other unique ROIs (1.2, 1.33, 1.92, Figure 4b). As a result, electrochemical conditioning is hypothesized to promote a homogeneous distribution of ionomer and carbon black along the cathode catalyst layer. Due to the binding nature of ionomer in the pristine catalyst layer, we attribute the changes in the I/C weight ratio to the redistribution of the newly formed free ionomer (Section 3.1) and ionomer stabilized carbon black during conditioning [63, 69]. Although the formation of free ionomer is hypothesized to compromise the structural cohesion of the catalyst layer (Section 3.1), its increased mobility enables redistribution during water uptake within the catalyst layer. In particular, free ionomer is proposed to migrate and accumulate near water‐dense platinum sites, resulting in similarly sized ionomer/carbon support groupings (observed in Figure S5, herein referred to as ionomer/carbon black synchronization) that lead to homogeneous I/C ratios. Moreover, we propose that the incipience of liquid water additionally accelerates carbon black interactions by swelling the ionomer and weakening the ionomer stabilized carbon (delamination of the ionomer from the platinum supported carbon black discussed in Section 3.1 for conditioned samples). As a result, the ionomer and carbon black particles agglomerate due to van der Waals interactions and form submicron agglomerates near similarly sized ionomer agglomerates [70]. This mechanism is supported by our observations of similarly sized ionomer and carbon black agglomerations (ionomer/carbon black synchronization) in conditioned catalyst layers (see Figure S5). In particular, Figure S5d shows substantial spatial overlap between similarly sized, relatively larger ionomer and carbon agglomerates within the catalyst layer. Likewise, Figure S5e,f exhibits groupings of ionomer and carbon black with comparable characteristic sizes at intermediate (Figure S5e) and smaller (Figure S5f) length scales. As a result, more spatially coordinated distributions form between ionomer and carbon black relative to pristine samples as a result of the formed agglomerations, contributing to enhanced I/C ratio uniformity across the analyzed ROIs.

**FIGURE 4:**
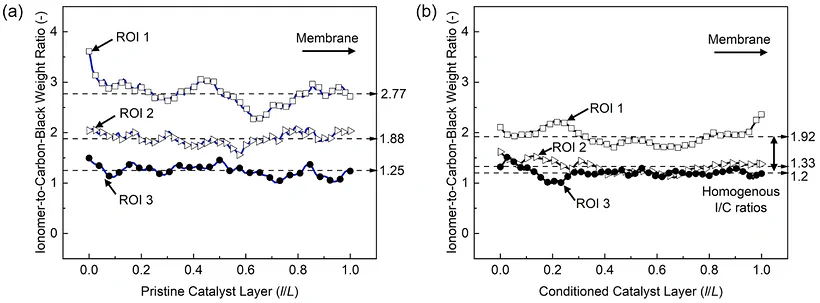
I/C weight ratio distributions for (a) pristine and (b) conditioned cathode catalyst layers as a function of the relative through‐plane distance (x), where *L* is the total thickness of each catalyst layer, and *l* is the local through‐plane position. The I/C weight ratios were quantified in three distinct ROIs along the cathode catalyst layer for each sample, with their respective averages indicated on the secondary *y*‐axis.

### 3.3 Ex Situ STXM Highlights Heterogeneous Porosity Distributions After Conditioning

We quantified the porosity distributions (described by Equation S9) for the pristine (Figure 5a) and conditioned CCMs (Figure 5b) to correlate the influence of electrochemical conditioning on the void volume of the cathode catalyst layer. Using Equation S9, the porosity is calculated as the difference between the total catalyst layer volume and volume occupied by the ionomer and carbon black. As a result, the trends depicted in Figure 5 are additionally representative of the relative changes between the ionomer and carbon black volume fraction distributions depicted in Figure 4 and share the same uncertainty parameters (Figures S1 and S3). Considering the relatively large spatial variations of the pristine and conditioned catalyst layer material distributions, it should be noted that the considered regions are primarily representative of local behavior rather than fully averaged catalyst layer porosity trends. Despite substantial changes to the I/C weight ratio distributions across different ROIs between the pristine and conditioned samples in Section 3.2, the relative porosity variations were found to be similar (Figure 5a,b). However, the porosity distributions for the conditioned CCMs exhibited significant fluctuations, evident by elevated root mean square error (RMSE) values across the three ROIs for the conditioned catalyst layer (0.038, 0.016, 0.028, Figure 5b) compared to the three ROIs for the pristine catalyst layers (0.02, 0.021, 0.016, Figure 5a). Despite an increase in I/C homogeneity for conditioned samples (Section 3.2), the increased porosity fluctuations are attributed to the evolution of water transport pathways during conditioning that are absent in the pristine samples [71, 72, 73, 74]. For conditioned CCMs, the maximum porosity was consistently observed at a distance of 0‐0.2 *L*/*L* (near the catalyst layer surface, Figure 5) due to an increased liquid water accumulation near the GDL during operation [42, 71, 75, 76, 77]. Moreover, the synchronized formation and redistribution of carbon black agglomerates (discussed in Section 3.2) could also contribute to the heterogeneous porosity distribution while maintaining a homogeneous I/C ratio distribution. Specifically, the migration of carbon black agglomerates toward the ionomer aggregates will result in local extrema in the porosity distribution and lead to a heterogeneous void space along the through‐plane direction.

**FIGURE 5:**
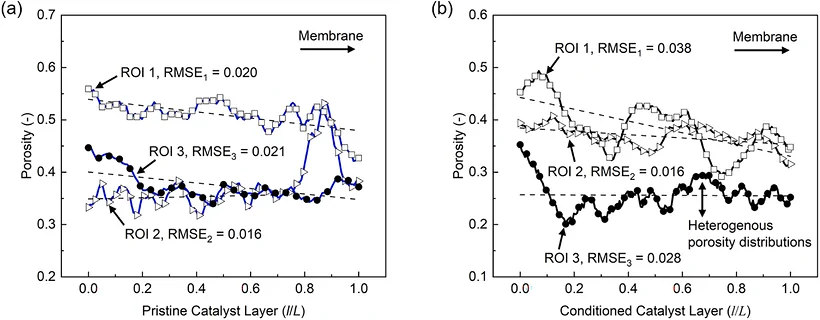
Through‐plane porosity distributions for (a) pristine and (b) conditioned cathode catalyst layers as a function of the relative through‐plane distance (x), where *L* is the total thickness of each catalyst layer, and *l* is the local through‐plane position. The porosity was quantified in the same three ROIs presented in Figure 4 for both samples, with their respective trendlines and RMSE values displayed.

### 3.4 In Situ STXM Reveals How Conditioned Membranes Contract under Fuel Cell Operating Temperatures

In this section, we reveal the effects of temperature on conditioned CCMs using in situ STXM. Conditioned samples were subjected to nanoscale membrane contraction upon heating to 60°C and 0% RH, as shown in Figure 6. Electrochemical conditioning leads to hydrated membranes which can contract in heated and dry environments due to the evaporation of liquid water [49, 78]. However, in ambient conditions, water is well retained due to the deposition of hydrophilic silica nanoparticles (SiO_2_) often added to assist with the hydration of PFSA‐based membranes [61, 79, 80, 81]. In contrast, since the pristine samples represented a dry membrane, they did not exhibit membrane contraction in heated conditions (Figure S6).

**FIGURE 6:**
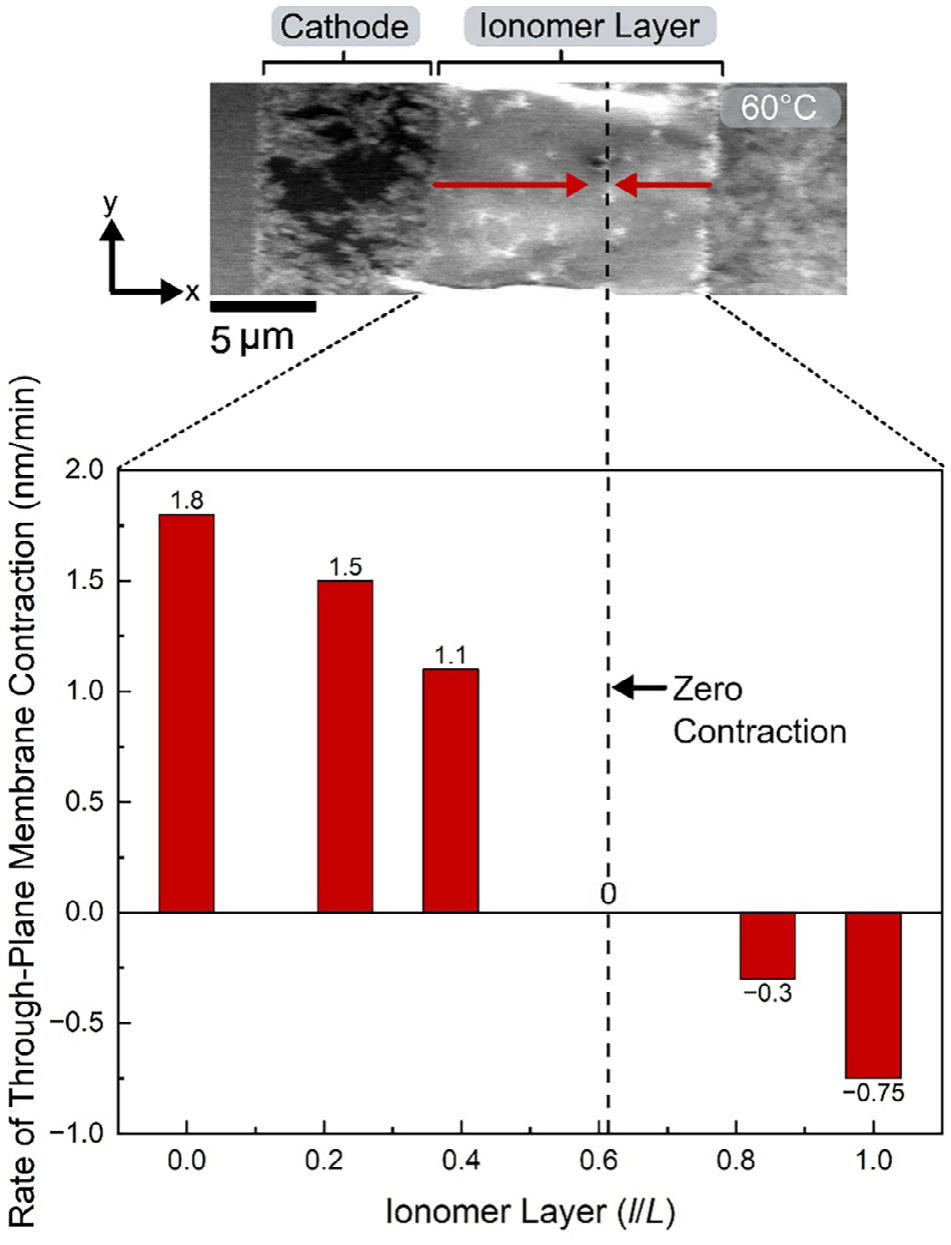
Rate of membrane contraction as a function of the relative through‐plane distance (x) across the ionomer layer, where *L* is the total thickness of the ionomer layer and *l* is the local through‐plane position. An asymmetrical contraction of the conditioned CCM was observed about the *x*‐distance.

The rate of nanoscale membrane contraction of a conditioned CCM was quantified over 159 min of constant temperature (60°C) imaging at the F K‐edge, and examined as a function of the through‐plane direction. A maximum local contraction was found near the membrane/catalyst layer interface (1.8 nm/min, Figure 6), whereas significantly less contraction was observed near the reinforcement layer (‐0.75 nm/min, Figure 6). The structurally degraded catalyst layer due to conditioning led to significant movement at the membrane/catalyst layer interface in comparison to the membrane/reinforcement interface, thus facilitating asymmetrical contraction of the CCM. Notably, at an *x*‐distance of 61% across the membrane through‐plane thickness (indicated by the vertical dashed line in Figure 6), membrane contraction was not detected.

As a result of the asymmetrical behavior, the membrane/catalyst layer interface is expected to have higher ohmic resistance and lower dynamic compression, resulting in inferior cell performance under dry conditions or in the event of membrane dehydration [48]. A total contraction of 2.55 nm/min was measured across the membrane, for a total approximate contraction of 0.405 µm over the considered timeframe (3.1% of membrane thickness). Thus, due to membrane contraction, when compressing a PEM fuel cell using fixed‐length compression (gaskets), a loss in assembly compression is expected when the cell is heated without sufficient hydration (such as during operational shutdown periods of the PEM fuel cell).

### 3.5 In Situ STXM Reveals Significant Catalyst Layer Damage Due to Membrane Contraction

Finally, the effects of membrane contraction on the nanoscale morphology of the catalyst layer were investigated using in situ STXM, as shown in Figure 7. We assumed that the initial void space area fraction (Figure 7a) was the result of tearing during ultramicrotomy, and membrane contraction exacerbated the catalyst layer damage after 159 min of constant heating (Figure 7b,c). Particularly, due to the catalyst layer adhesion (lamination) to the membrane, membrane contraction led to an increase in the average void space area fraction in the catalyst layer (by 9.3%) at 60°C and dry conditions (Figure 7c).

**FIGURE 7:**
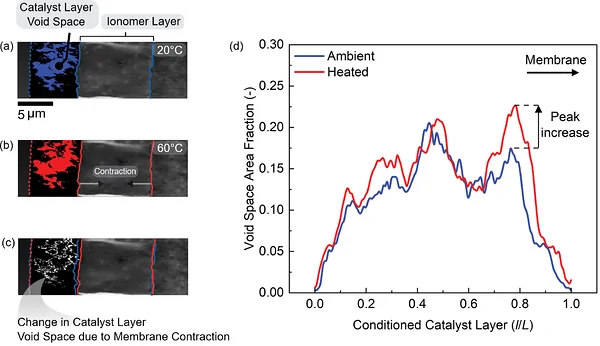
STXM imaging revealing the catalyst layer deformation during dry, heated in situ conditions: (a) Binarized image of the conditioned CCM void space initially subjected to 60°C and dry conditions (blue), (b) binarized image of the resulting catalyst layer void space after 159 min of constant temperature heating (red), (c) binarized image of the difference in void space between (a) and (b) (white) due to membrane contraction, and (d) void space area fraction distribution for the ambient (a) and heated (b) catalyst layer as a function of the relative through‐plane distance (x), where *L* is the total thickness of the catalyst layer and *l* is the local through‐plane position.

Moreover, the peak rate of deformation in the catalyst layer was observed at the catalyst layer/membrane interface (Figure 7d), where, in Section 3.4, the maximum rate of membrane deformation was quantified. The void space area fraction distributions revealed that membrane contraction promotes the formation of additional void space throughout the catalyst layer, and gradually increases toward the catalyst layer/membrane boundary. We propose that the increase in void space along the *x*‐direction resulted from the asymmetrical membrane contraction observed in Section 3.3, ultimately resulting from the inferior structural integrity of the conditioned catalyst layer. Thus, in the event of membrane contraction, cyclic mechanical stresses are expected to result in the structural degradation of the catalyst layer nanostructure over time, particularly near the catalyst layer/membrane interface. Notably, since the considered CCM was in an uncompressed state (free‐space), the degree of catalyst layer strain observed during the 159‐minute constant heating test is not necessarily representative of a CCM assembled in a fuel cell. Rather, the observed catalyst layer tearing is indicative of the possibility of internal stresses acting on the catalyst layer nanostructure in the event of membrane contraction/expansion. As a result, we expect that cyclic membrane expansion/contraction may result in significant catalyst layer damage and interfacial contact losses near the membrane after extended periods of operation, resulting in elevated ohmic overpotentials and PEM fuel cell performance degradation.

## 4 Conclusions

We quantified the effects of conditioning and temperature on PEM fuel cell CCMs using a combination of ex situ and in situ STXM. Catalyst layer degradation was caused by conditioning, which can be primarily attributed to electrochemically induced ionomer degradation. Our work also explains an important beneficial outcome of conditioning whereby it promotes homogeneous I/C weight ratio distributions along the cathode catalyst layer, which we attribute to the formation of carbon black agglomerates upon the incipience of liquid water. However, conditioned CCMs exhibited a heterogeneous porosity distribution, which we attribute to regions of high and low water saturation in the catalyst layer.

Furthermore, in contrast to pristine samples, conditioned membranes dynamically contract when subjected to heated and dry conditions. The rate of membrane contraction was spatially dependent on the through‐plane membrane position, with the highest rate of contraction observed at the membrane/catalyst layer interface (1.8 nm/min). Even though conditioning leads to performance improvements in the short‐term, conditioning may compromise the mechanical strength of the catalyst layer. As a result, in the event of membrane expansion/contraction during temperature cycling of PEM fuel cells, CCMs may experience cyclic fatigue over time. Thus, to enhance catalyst layer durability, we encourage the further investigation of operation strategies that mitigate CCM swelling and shrinkage, thereby improving PEM fuel cell performance and durability.

## Author Contributions

S.L. performed conceptualization, methodology, validation, formal analysis, resources, data curation, writing (original draft, review, and editing), and visualization. H.P. performed conceptualization, methodology, validation, resources, data curation, and writing (review and editing). J.W. performed methodology, resources, data curation, writing (review and editing). S.Y. performed methodology, resources, data curation, writing (review and editing). A.B. performed conceptualization, methodology, resources, data curation, writing (review and editing), supervision, project administration, and funding acquisition.

## Conflicts of Interest

The authors declare no conflicts of interest.

## Supporting information




**Supporting File**: advs77009‐sup‐0001‐SuppMat.docx.

## Data Availability

The data that support the findings of this study are available from the corresponding author upon reasonable request.
