# Supplementary material for: Revealing the Nanoscale Morphology Changes of Polymer Electrolyte Membrane Fuel Cell Catalyst Layers due to Conditioning
Source: Adv Sci (Weinh). 2026 Aug 10;13(60):e77009. doi: 10.1002/advs.77009 (PMC13455836; doi:10.1002/advs.77009)
Supplement: Supplementary file 1 [file ADVS-13-e77009-s001.docx]

**Supporting Information**

For

**Revealing the nanoscale morphology changes of polymer electrolyte membrane fuel cell catalyst layers due to conditioning**

S. Lytle^a^, H.B. Parmar^a^, J. Wang^b^, S. Yamashita^c^, and A. Bazylak^a*^

^a^ Bazylak Group, Department of Mechanical & Industrial Engineering, Faculty of Applied Science and Engineering, University of Toronto, Toronto, Ontario M5S 3G8, Canada

^b^ Canadian Light Source Inc., Saskatoon, Saskatchewan S7N 2V3, Canada

^c^ Photon Factory, Institute of Materials Structure Science,

High Energy Research Organization, 1-1 Oho, Tsukuba, Ibaraki 305-0801, Japan

Contents

[**1.0 Supplementary Methods**	3](#_Toc233459411)

[**2.0 Experimental Section**	19](#_Toc233459412)

# **1.0 Supplementary Methods**

After data collection, the energy stacks were processed using the spectromicroscopy analysis software aXis2000^1^. During the energy stack acquisition, minor spatial shifts are inherent to the STXM instrumentation. To correct for image misalignment during imaging, energy stack images were aligned using a Fourier cross-correlation procedure^2^ outlined in Equation S1.

$$c_{k}=\mathcal{F}^{-1}\left\{ \mathcal{F}\left( E_{1} \right)\cdot\left[ \mathcal{F}\left( E_{k} \right) \right]^{*} \right\}, k \in\left( 1, n \right)$$

(S1)

Where $c_{k}$ is the cross-correlation function of the first image ($E_{1}$) with respect to the $k^{th}$ ($E_{k}$) image, and $\mathcal{F}\left( E_{1} \right)$ and $\mathcal{F}\left( E_{k} \right)$ are the intensity frequency domains of the first and last image, respectively. The cross-correlation function was found by converting the first and $k^{th}$ images into frequency domains ($\mathcal{F}\left( E_{1} \right)$ and $\mathcal{F}\left( E_{k} \right)$, respectively) via the Fourier transform. By taking the dot product of the complex conjugate of the $k^{th}$ image Fourier transform function $(\left[ \mathcal{F}\left( E_{k} \right) \right]^{*})$ by the Fourier transform function of the first image ($\mathcal{F}\left( E_{1} \right)\cdot\left[ \mathcal{F}\left( E_{k} \right) \right]^{*}$), a frequency domain highlighting only the shift in frequencies was obtained. Subsequently, by taking the inverse of the dot product, the frequency shifts were converted back to a spatial domain, where the cross-correlation position was quantified. Finally, the $k^{th}$ image was shifted by the difference in position between the cross-correlation location of the $k^{th}$ image and center of the first image to align the image stack.

Subsequently, the X-ray absorption in the images of each energy stack was quantified using the optical density ($OD$). Optical density images ($OD\left( x_{i},y_{j} E_{k} \right)$ [-]) were obtained by logarithmically subtracting the transmission signal ($I_{s}(x_{i},y_{j}, E_{k})$ [-]) through each pixel ($x_{i},y_{j}$[-]) of an image at energy ($E_{k}$[eV]) by the background transmission signal ($I_{0}(E_{k}$*)* [-]) using the Beer-Lambert law, given by Equation S2.

$OD\left( x_{i},y_{j} E_{k} \right)=ln\frac{I_{0}\left( E_{k} \right)}{I_{s}\left( x_{i},y_{j},E_{k} \right)}$ $i \in\left( 1, n_{x} \right), j \in\left( 1, n_{y} \right), k \in\left( 1, n_{E} \right)$

(S2)

Where $n_{x}$ and $n_{y}$ are the total number of pixels in the *x*-direction and *y*-direction, respectively, and $n_{E}$ is the total number of energies compiling the stack. For all transmission-based images, Equation S2 was applied to each pixel in the *x*-direction and *y*-direction, the compilation of which is the optical density-based energy stack. With this, the average X-ray absorption spectra ($\bar{OD}(E_{k})$) for Nafion and carbon black support were calculated for the membrane regions of each CCM and carbon black powder sample, respectively, defined by Equation S3.

$$\bar{OD}\left( E_{k} \right)=\frac{\sum_{i=1}^{n_{i}} \sum_{j=1}^{n_{j}} OD(x_{i},y_{j},E_{k})}{n_{i}n_{j}}$$

(S3)

Where the number of pixels in the *x*-direction and *y*-direction of the selected region in the energy stack ROI are represented by $n_{i}$ and $n_{j}$. Thereafter, to obtain the Nafion and carbon black thickness maps [nm] from the optical density image stacks, the average spectra for Nafion and carbon black (Equation S3) were normalized to the theoretical X-ray absorption corresponding to a 1 nm thick sample. The normalized X-ray absorption spectra were then used to calculate the thickness distributions [nm] of the respective species in each sample by dividing the total absorption measured in each pixel [OD] by the normalized spectra [OD/nm] for Nafion and carbon black^2,3^.

To find the normalized spectra for Nafion and carbon black, a normalizing scaling factor ($\alpha$ [-]) was first calculated in Equation S4:

$$\alpha=\frac{\bar{OD}(E_{1})}{\mu(E_{1})}=\frac{\bar{OD}(E_{n_{E}})}{\mu(E_{n_{E}})}$$

(S4)

Where, for a respective species, ($\bar{OD}(E_{1})$ [-]) is the average species absorption at the first energy ($E_{1}$), ($\bar{OD}(E_{n_{E}})$ [-]) is the average species absorption at the final energy ($E_{n_{E}}$), ($\mu(E_{1})$ [nm^-1^]) is the theoretical absorption per nm at the first energy ($E_{1}$), and ($\mu(E_{n_{E}})$ [nm^-1^]) is the theoretical absorption per nm at the final energy ($E_{n_{E}}$). The resulting factor, $\alpha$ [nm], was calculated for both Nafion and carbon black spectra, and employed to normalize each respective spectrum $OD1\left( E_{k} \right)$ [OD/nm] by performing the following operation, as per Equation S5.

$$OD1\left( E_{k} \right)=\frac{\bar{OD}(E_{k})}{\alpha}, k\in\left( 1, n_{E} \right)$$

(S5)

Where $\bar{OD}(E_{k})$ [OD] is the average calculated absorption spectrum of Nafion or carbon black, and $\alpha$ [nm] is the respective normalizing factor to obtain the normalized spectrum ($OD1\left( E_{k} \right)$).

Thereafter, the thickness of Nafion and carbon black in each pixel ($x_{i}, y_{j}$ [-]) of the CCM was determined through an iterative fitting process in aXis2000 (Equation S6) using the normalized Nafion and carbon black reference spectrum $OD1\left( E_{j} \right)$ calculated from Equation S5.

$OD\left( x_{i},y_{j},E_{k} \right)=t_{l}\left( x_{i},y_{j} \right)\cdot OD1_{l}\left( E_{k} \right)+\theta\left( P_{i},E_{j} \right)+\kappa\left( x_{i},y_{j} \right)$ $l\in\left( 1, n_{s} \right)$

(S6)

Where, $t_{l}\left( x_{i}, y_{j} \right)$ [nm] is the thickness of a particular species, $OD1(E_{k})$ [nm^-1^] is the normalized spectra corresponding to the respective species, and $n_{s}$ [-] represents the number of independent species being probed. Moreover, $\theta\left( P_{i},E_{j} \right)$ [-] represents the residual absorption error accumulated during the iterative fitting procedure, and $\kappa\left( x_{i},y_{j} \right)$ [-] accounts for the baseline (constant) X-ray adsorption of the ROI. Using a least squares fit model, the collected catalyst layer spectrum (Equation S3) is fitted with a theoretical catalyst layer spectrum as a unique combination of the reference spectra. The resulting spectrum is known as the fitted spectrum, whose difference with the collected catalyst layer spectrum is the residuals of the fit and indicates the uncertainty of the results. The fitted and reference spectra, along with their corresponding residuals, are provided for each ROI analyses below in the Supplementary Information.”

Furthermore, to particularly distinguish carbon in nafion ionomer from carbon black, we employed X-ray absorption spectroscopy alongside least squares fit model analyses^3,4^. Since carbon black and nafion contain unique carbon bonds (such as $\pi^{*}(C=C)$ for carbon support at 285.5 eV and $\sigma^{*}\left( C-F \right)||$ bonds for nafion above 293 eV (particular 296 eV for undamaged nafion))^5,6^ (see Figure S1 and S3), their X-ray absorption spectra are unique and can ultimately be used as a material “fingerprint” to distinguish between ionomer and carbon black in the catalyst layer^3^. Since the catalyst layer contains a mixture of carbon black and nafion, the resulting X-ray absorption spectrum of the catalyst layer is a combination of the carbon black and nafion X-ray absorption spectra. Using the intrinsic X-ray absorption spectra of pure carbon black and nafion at the carbon K-edge (reference spectra), the reference spectra can be scaled to match the collected catalyst layer spectrum using a least squares fit model. The uncertainty of the least squares fit model is quantified using the $\chi^{2}$ parameter, and visualized by the resulting residual spectrum calculated as the difference between the catalyst layer spectrum and least squares fit model spectrum. A $\chi^{2}$ value greater than one is generally considered a poor fit^7^.

The raw carbon black and nafion reference spectrum, least squares fit model spectrum, and residual spectrum for each ROI corresponding to Figure 4 and Figure 5 in the manuscript are shown in Figure S1 and Figure S3, respectively. In addition, the $\chi^{2}$ parameter is given for each ROI to quantify the uncertainty of each fit. For the pristine analyses (Figure S1), the $\chi^{2}$ parameter for ROI 1, ROI 2, and ROI 3 is 0.631 (Figure S1), 0.104 (Figure S1), and 0.167 (Figure S1), respectively. Since a lower $\chi^{2}$ value represents a better goodness of fit, ROI 1 ($\chi^{2}=0.631$, Figure S1) quantitatively exhibits a relatively poorer goodness of fit. However, imperfect $\chi^{2}$ values are expected in overlapping low-Z species, in particular when experimental noise and material signal is low. In the case of ROI 1, the highest residual is apparent near the characteristic peak of carbon black ($285 eV$, Figure S1), resulting in an elevated $\chi^{2}$ value. The original data showcasing the pristine volume fraction distributions of nafion and carbon black is given in Figure S2, and demonstrates that ROI 1 exhibits the lowest concentration of the carbon black species (volume fraction between 10-15%, Figure S2) compared to the other ROIs. This is anticipated to result in a high noise-to-signal ratio that enlightens the high $\chi^{2}$ associated with ROI 1, additionaly supported by the increased residual intensity near the carbon black characteristic peak in Figure S1(a). However, despite a lower carbon black signal, the major spectral features are still captured in the least squares fit model in Figure S1(a) while the residual intensity remains small compared to the overall signal, supporting the validity of the component differentiation. Moreover, Figure S3 demonstrates a consistently acceptable $\chi^{2}$ value across the three ROIs, with the processed nafion and carbon black volume fraction distribution showcased in Figure S4.

The original volume fraction distribution data for nafion and carbon black across the catalyst layer supports our findings that conditioning leads to more homogenous I/C ratios due to the relative reorganization between nafion and carbon black. Particularly for the pristine samples, the I/C ratio is observed to vary drastically along the three ROIs (Figure 4 in manuscript), with no patterning correlation between the nafion and carbon black volume fraction distributions (Figure S2). In contrast, the conditioned samples are observed to exhibit homogenous I/C ratios across the three ROIs (Figure 5 in manuscript), with observable synchronization between the nafion and carbon black volume fraction distributions. The observed synchronization is attributed to the relative reorganization of the nafion and carbon black during electrochemical conditioning that aids in the normalization of the I/C ration along the length of the catalyst layer. In addition, the locations of the three ROIs for both the pristine and conditioned samples can be found in the description of Figure S5.

(a) ROI 1 Pristine
(b) ROI 2 Pristine
(c) ROI 3 Pristine

**Figure S1:** Least squares fit model analyses for the ionomer-to-carbon black ratio (I/C) investigation originating from Figure 4 of the manuscript, where (a) ROI 1, (b) ROI 2, and (3) ROI 3. For each ROI, the catalyst layer spectrum is shown, alongside the least squares fit model that was constructed to match the catalyst layer spectrum using the carbon black and nafion reference spectra. The uncertainty of the least squares fit model is quantified through the respective $\chi^{2}$. Notably, ROI 1 exhibits the highest $\chi^{2}$ ($\chi^{2}=0.631$), indicating a poorer goodness of fit between the experimental catalyst layer spectrum and least squares fit model spectrum. However, the fit quality remains acceptable as $\chi^{2}<1$. Moreover, the residual intensity is small compared to the optical density signal of the catalyst layer spectrum and remains close to zero along the entirety of the energy spectrum. Despite the larger $\chi^{2}$ compared to ROI 2 and ROI 3, the model still captures the characteristic absorption edge peaks.

(a) ROI 1 Pristine
(b) ROI 2 Pristine
(c) ROI 3 Pristine

**Figure S2:** Volume fraction distributions of nafion and carbon black in the pristine catalyst layer used to calculate the ionomer-to-carbon black ratio (I/C) from Figure 4 of the manuscript, where (a) ROI 1, (b) ROI 2, and (3) ROI 3. The x-axis is a normalized length scale across the through-plane dimension of the catalyst later, where 0.0 is approximated near the PS/catalyst layer interface, and 1.0 is approximated as the catalyst layer/membrane interface.

(a) ROI 1 Conditioned
(b) ROI 2 Conditioned
(c) ROI 3 Conditioned

**Figure S3:** Least squares fit model analyses for the ionomer-to-carbon black ratio (I/C) investigation originating from Figure 5 of the manuscript, where (a) ROI 1, (b) ROI 2, and (3) ROI 3. For each ROI, the catalyst layer spectrum is shown, alongside the least squares fit model that was constructed to match the catalyst layer spectrum using the carbon black and nafion reference spectra. The uncertainty of the least squares fit model is quantified through the respective $\chi^{2}$.

(a) ROI 1 Conditioned
(b) ROI 2 Conditioned
(c) ROI 3 Conditioned

**Figure S4:** Volume fraction distributions of nafion and carbon black in the conditioned catalyst layer used to calculate the ionomer-to-carbon black ratio (I/C) from Figure 5 of the manuscript, where (a) ROI 1, (b) ROI 2, and (3) ROI 3. The x-axis is a normalized length scale across the through-plane dimension of the catalyst later, where 0.0 is approximated near the PS/catalyst layer interface, and 1.0 is approximated as the catalyst layer/membrane interface.

Furthermore, the average thickness of a particular species ($\bar{t}_{l}$) in a given ROI was determined as follows in Equation S7:

$$\bar{t}_{l}=\frac{\sum_{i=1}^{n_{i}} \sum_{j=1}^{n_{j}} t_{l}(x_{i},y_{j})}{n_{i}n_{j}}$$

(S7)

Where $t_{l}(x_{i},y_{j})$ is the thickness of a particular species through a particular pixel, and ($n_{i}n_{j}$) is the total number of pixels enclosing the selected region of interest.

Moreover, the volume fraction distribution ($\phi(x)$ [-]) of ionomer and carbon black in the through-plane direction (*x*-distance) for each catalyst coated membrane was quantified as follows in Equation S8:

$$\phi_{l,y}\left( x \right)=\frac{\bar{t}_{y}(x)}{\bar{t}_{m}}$$

(S8)

Where $\bar{t}_{y}\left( x \right) [\mathrm{nm}]$ is the average thickness of the probed species in the *y*-direction as a function of the through-plane direction (*x*-distance), and $\bar{t}_{m}$ [nm] is the calculated thickness of the membrane. Notably, the area fractions of ionomer and void space of pristine and conditioned catalyst layers were quantified via manual signal intensity thresholding (masking). Regions for masking were selected based on areas with a ±5% similarity in $I_{0}$ at a particular energy. Moreover, the area of the masked image, consisting of void space or ionomer, was then normalized with respect to the total catalyst layer area to calculate the area fraction. Moreover, the volume fraction distribution of porosity was determined by employing Equation S9.

$$\emptyset_{p}\left( x \right)=1-\emptyset_{i}\left( x \right)-\emptyset_{c}\left( x \right)$$

(S9)

Where $\emptyset_{p}\left( x \right)$ [-] is the porosity distribution as a function of the *x*-direction, $\emptyset_{i}\left( x \right)$ [-] is the volume fraction distribution of ionomer as a function of the *x*-direction, and $\emptyset_{c}\left( x \right)$ [-] is the volume fraction distribution of carbon black as a function of the *x*-direction.

Finally, the ionomer-to-carbon-black weight ratio was calculated throughout each CCM. The ionomer-to-carbon-black weight ratio was quantified by dividing the mass of ionomer to the mass of carbon support, simplified in Equation S10.

$$\emptyset(x)=\frac{\rho_{i}\bar{t}_{i,y}(x)}{\rho_{c}\bar{t}_{c,y}(x)}$$

(S10)

Where $\rho_{i}$ and $\rho_{c}$ are the densities of ionomer ($1.98 g/cm^{3}$) and carbon black ($2 g/cm^{3}$), respectively, and $\bar{t}_{i,y}(x)$ and $\bar{t}_{c,y}\left( x \right)$ are the computed thickness distributions of ionomer and carbon black, respectively, as a function of the *x*-direction.

# **2.0 Experimental Section**

**Figure S5:** Material distributions of nafion (green), carbon black (red), and overlapping nafion and carbon black (yellow) of the pristine (a)-(c) and conditioned (d)-(f) ROIs. Pristine ROIs (a)-(c) are observed to exhibit independent nafion and carbon black distribution behaviors, ultimately resulting in larger I/C ratio variances. In contrast, the conditioned ROIs (d)-(f) demonstrate natural clustering/agglomeration of carbon black and nafion after electrochemical conditioning that evidently increase the I/C ratio uniformity across the ROIs. For both the pristine and conditioned analyses, ROI 2 and ROI 3 were acquired from the same sample and were separated by approximately 35 µm. ROI 1 was acquired from a different sample to minimize the influence of potential sampling bias.

**Figure S6:** In-situ STXM showcasing the difference between heating of a conditioned sample (a) and (b) VS a pristine sample (c) and (d) after 159 minutes at 60°C. The membrane of the conditioned sample shows a dramatic contraction during heating due to membrane dehydration, while minimal membrane movement is observed for the dry pristine sample.

**References as numbered in this report**

1.	Hitchcock, A. P. Analysis of X-ray images and spectra (aXis2000): A toolkit for the analysis of X-ray spectromicroscopy data. *J. Electron Spectros. Relat. Phenomena* **266**, 147360 (2023).

2.	Jacobsen, Wirick, Flynn & Zimba. Soft X‐ray spectroscopy from image sequences with sub‐100 nm spatial resolution. *J. Microsc.* **197**, 173–184 (2000).

3.	Marcus, M. A. Data analysis in spectroscopic STXM. *J. Electron Spectros. Relat. Phenomena* **264**, 147310 (2023).

4.	Susac, D., Berejnov, V., Hitchcock, A. P. & Stumper, J. STXM Characterization of PEM Fuel Cell Catalyst Layers. *ECS Trans.* **50**, 405–413 (2013).

5.	Martens, I., Melo, L. G. A., Wilkinson, D. P., Bizzotto, D. & Hitchcock, A. P. Characterization of X-ray Damage to Perfluorosulfonic Acid Using Correlative Microscopy. *The Journal of Physical Chemistry C* **123**, 16023–16033 (2019).

6.	Susac, D., Berejnov, V., Hitchcock, A. P. & Stumper, J. STXM Study of the Ionomer Distribution in the PEM Fuel Cell Catalyst Layers. *ECS Trans.* **41**, 629–635 (2011).

7.	Morgan, I. L. & Saleh, O. A. Tweezepy: A Python package for calibrating forces in single-molecule video-tracking experiments. *PLoS One* **16**, e0262028 (2021).
